# *Wickerhamomyces anomalus* as a biocontrol agent against *Aspergillus clavatus* and patulin in apples

**DOI:** 10.1038/s41598-026-57297-5

**Published:** 2026-06-19

**Authors:** Emelin Leandro Rodrigues, Enikő Horváth-Szanics, Olívia Csernus, Zsolt Zalán, Ildikó Bata-Vidács

**Affiliations:** 1 Department of Bioengineering and Fermentation Technology, Hungarian University of Agriculture and Life Sciences, Budapest, Hungary; 2 Department of Food Chemistry and Analytics, Institute of Food Science and Technology, Hungarian University of Agriculture and Life Sciences, Budapest, Hungary; 3 Department For Chemical Safety and Competent Authorities, National Center for Public Health and Pharmacy, Budapest, Hungary; 4 Centre for Research and Development, Eszterházy Károly Catholic University, Eger, Hungary

**Keywords:** Patulin, *Wickerhamomyces anomalus*, *Aspergillus clavatus*, Toxin removal, Biocontrol, Yeast adsorption, Postharvest disease control, Food safety, Biotechnology, Microbiology

## Abstract

Apple is a widely consumed fruit, but its quality can be compromised by mycotoxins such as patulin, produced mostly by *Penicillium expansum*, but also by the less-studied *Aspergillus clavatus*. This toxin poses a health risk, requiring effective control strategies. Biocontrol, particularly using yeasts, has gained attention as a promising method. In this study, we assessed the potential of different yeast strains to inhibit the growth of the patulin (PAT)-producing *A. clavatus* B9/6 and to remove patulin. While *Saccharomyces cerevisiae*, previously reported as an antagonist of *P. expansum*, showed no inhibitory effects on *A. clavatus*, all 13 *Wickerhamomyces anomalus* strains studied exhibited antifungal activity to varying degrees. Among them, strains 01499, 0961, 0170, and 01655 showed the strongest inhibition. Notably, *W. anomalus* 01499 not only inhibited fungal growth but also removed patulin, reducing the toxin by 71% within 24 h and achieving complete removal after 48 h under the tested experimental conditions (10 ppm patulin in apple juice). In vivo assays in apples showed that pre-inoculation with *W. anomalus* 01499 strongly suppressed fungal infection, whereas delayed application resulted in more severe symptoms, emphasizing the importance of early intervention. Mechanistic studies revealed that patulin removal occurred primarily through cell wall binding (11%) and intracellular components (including enzymes) (33%), while no contribution from extracellular enzyme activity was detected. These results suggest that *W. anomalus* 01499 is a promising candidate for the biocontrol of *A. clavatus* and for reducing patulin contamination in apples. This study provides further evidence supporting the potential role of *W. anomalus* in patulin removal and fungal biocontrol.

**Supplementary Information:**

The online version contains supplementary material available at https://doi.org/10.1038/s41598-026-57297-5.

## Introduction

Apples (*Malus domestica*) are among the most widely produced and consumed fruits worldwide, valued for their nutrition, economic relevance, and versatility, with global production exceeding 95 million tons annually according to FAO statistics ^1^. However, their susceptibility to fungal contamination, particularly by the mycotoxin patulin (PAT), poses a major food safety concern. This toxin is mainly produced by *Penicillium*, *Aspergillus*, and *Byssochlamys* spp. ^2^. Patulin was first investigated in the 1940s for its antimicrobial properties ^3^. However, it was later recognized as a food contaminant associated with adverse health effects, including genotoxicity, immunotoxicity, and neurotoxicity, primarily affecting the kidneys, immune system, and intestinal tissues ^4^. Research on patulin contamination has primarily focused on *Penicillium* species, which are the main contributors to contamination in fruits and their derivatives ^5,6^. In contrast, the role of *Aspergillus* in patulin production remains underexplored. Recent studies indicate that certain *Aspergillus* strains may also contribute to patulin contamination, underscoring a research gap in mycotoxin studies ^7,8^. Addressing this gap offers an important opportunity to investigate *Aspergillus* as a potentially overlooked source of patulin, motivating our focus on this genus in the present study.

Due to its health risks, regulatory agencies have set strict limits for PAT in foods. The World Health Organization (WHO) recommends a provisional maximum tolerable intake of 0.4 µg/kg body weight per day, while the European Food Safety Authority (EFSA) sets maximum permitted concentrations of 50 µg/kg for juices, 25 µg/kg for solid apple products, and 10 µg/kg for products for infants and young children ^9^. Brazil and China adopt 50 µg/kg for juices, with no limits for other apple products ^10^.

Approaches for patulin control fall into two main categories: pre-harvest and post-harvest strategies. Pre-harvest methods focus on preventing fungal growth in orchards, whereas post-harvest strategies include measures applied during storage, handling, and processing to inhibit fungal development and limit patulin contamination in both fresh apples and derived food products ^11^. Recently, biocontrol approaches employing microorganisms, enzymes, essential oils, and plant extracts have gained increasing attention as environmentally friendly alternatives for patulin control and degradation. Among these, microbial degradation has shown particular promise ^12,13^. In addition to bacterial agents, several yeast species have also been identified as effective biocontrol candidates against patulin.

*Wickerhamomyces anomalus* (formerly *Pichia anomala*) stands out due to its resilience in harsh environments, including low pH and high concentrations of salt and sugar ^14^. This adaptability makes *W. anomalus* particularly suitable for industrial applications.

In food processing, *W. anomalus* contributes to flavor enhancement in bakery goods; it is also used in winemaking and fermented beverages, often co-fermenting with *Saccharomyces cerevisiae*, improving aroma complexity and preventing microbial spoilage through the production of killer toxins ^15,16^.

The ability of *W. anomalus* to produce volatile organic compounds, biosurfactants, and killer toxins supports its potential as a safe biocontrol agent against mycotoxin-producing fungi. This study evaluated its antifungal activity against *A. clavatus* and its capacity for patulin removal, contributing additional evidence for the potential of *W. anomalus* in patulin mitigation ^17^.

## Materials and methods

### Strains

The *Aspergillus clavatus* B9/6 strain was previously isolated from apples and identified by our group, with patulin production confirmed ^18^. Three groups of yeasts were investigated: commercial strains and *Saccharomyces* and *Wickerhamomyces* strains obtained from the Hungarian National Collection of Agricultural and Industrial Microorganisms (NCAIM). The tested strains are listed in Table 1. *Candida robusta* and *S. willianus* were reclassified as *S. bayanus* and *S. cerevisiae*, respectively.

**Table 1:** Yeast strains screened for antagonistic activity against *A. clavatus* B9/6. Commercial strains are listed in the first column, while strains obtained from the Hungarian National Collection of Agricultural and Industrial Microorganisms (NCAIM) are grouped according to species.

Commercial strains	*Saccharomyces* strains	*Wickerhamomyces* strains
HD A 54^®^	*S. cerevisiae* 0019	*W. anomalus* 00170
HD 562^®^	*S. cerevisiae* 00164	*W. anomalus* 00213
HD S135^®^	*S. cerevisiae* 00204	*W. anomalus* 00367
GV S107^®^	*S. cerevisiae* 00205	*W. anomalus* 00459
Uvaferm^®^	*S. cerevisiae* 00208	*W. anomalus* 00545
Uvaferm Danstil A^®^	*S. cerevisiae* 00210	*W. anomalus* 00548
Uvaferm 228^®^	*S. cerevisiae* 00225	*W. anomalus* 00717
Jazz^®^	*S. cerevisiae* 00677	*W. anomalus* 00927
Safcider AS-2/ AB-1/ TF- 6^®^	*S. cerevisiae* 0151	*W. anomalus* 00935
Craft series MO2^®^	*S. cerevisiae* 0171	*W. anomalus* 00961
Safcider^®^	*S. cerevisiae* 0206	*W. anomalus* 01109
Safbrew LA-01^®^	*S. cerevisiae* 0220	*W. anomalus* 01499
Spiriferm^®^	*S. cerevisiae* 0223	*W. anomalus* 01655
Oenoferm Xtreme/X-thiol^®^	*S. cerevisiae* 0224	
CM^®^	*C. robusta*	
Flavia^®^	*S. willianus*	
	*S. bayanus* 0120	

(Commercial strains were classified according to manufacturer specifications; all commercial strains are classified as *S. cerevisiae* according to manufacturer information, except Safcider AB-1, which is a *S. bayanus* × *S. cerevisiae* hybrid).

### Selection of yeasts for potential growth inhibition of *A. clavatus* B9/6

A first preliminary screening test was done with all the listed yeast strains, spores of *A. clavatus* B9/6 were inoculated from an initial suspension of 10^7^ conidia/mL into malt extract agar (MEA) (Malt Extract Agar, VWR), cooled to 45 °C to achieve a final concentration of 10^3^ conidia/mL. The medium was poured into sterile Petri dishes, allowed to solidify, and streaked at the center with yeast biomass grown on PDA at 25 °C for 24 h. Control plates containing only the fungal inoculum were included in parallel. Plates were incubated at 25 °C and evaluated after 48 h and 7 days. Fungal growth inhibition was assessed qualitatively by comparing mold development in the vicinity of the yeast streak with that observed on control plates containing only the fungal inoculum.

Additionally, a second experiment was performed with those yeasts which show fungal growth inhibition in the preliminary screening test. A central streak of *A. clavatus* B9/6 (10⁷ conidia/mL) was made on PDA, with parallel streaks of yeast (10⁸ CFU/mL) placed on either side at a distance of 20 mm. Control plates containing only the fungal inoculum were included in parallel. Plates were incubated at 25 °C and evaluated after 48 h and 7 days. Fungal growth was assessed as the appearance of colonies outside the initial inoculation zone. To provide a semi-quantitative evaluation of antifungal activity, strains were classified according to the number of colonies detected beyond the inoculation zone as follows: score 0, more than five colonies; score 1, three to five colonies; score 2, one to two colonies; and score 3, no colonies detected beyond the inoculation zone. Strains with the highest scores were selected for subsequent experiments.

### Patulin removal by yeasts

Yeast strains showing the strongest inhibition of mold growth were selected for the patulin removal experiment, as the study aimed to identify candidates with a combined biocontrol effect, including both suppression of fungal development and reduction of patulin contamination. One milliliter with a concentration of 10^8^ CFU/mL of each strain was inoculated into 20 mL of commercial apple juice (100% pasteurized apple juice purchased from a local supermarket) spiked with 10 ppm of patulin (LGC, Toronto Research Chemicals, Canada), a concentration selected to ensure reliable analytical detection and accurate quantification of toxin removal under controlled laboratory conditions. The inoculated flasks were incubated at 25 °C under constant agitation (150 rpm) (NB205V, N-Biotek, South Korea) for 24 h. Additionally, a control flask without yeast was included. Following incubation, patulin was extracted from the samples then analyzed using HPLC. The results were compared to the initial patulin levels to assess the PAT removal efficiency of each *W. anomalus* strain.

### Kinetics of patulin removal

For the PAT removal kinetics studies, 1 mL at a concentration of 1 × 10^8^ CFU/mL of the selected yeast strains was inoculated into 20 mL of apple juice spiked with 10 ppm of patulin. The cultures were incubated at 25 °C with constant agitation for 48 h. Samples of 2 mL were collected throughout the 48-hour period at 0, 4, 8, 20, 28, 30, and 48 h, including the initial time point to assess patulin removal over time. Sampling points were selected to monitor both the early rapid removal phase and the later stabilization period of patulin concentration. A control flask without yeast was included to evaluate the natural degradation of patulin in the medium. Patulin was extracted, and the concentrations quantified *via* HPLC were plotted against time.

### In vivo inhibition of *A. clavatus* B9/6 by *W. anomalus*

To evaluate the inhibition of *A. clavatus* B9/6 by *W. anomalus* 01499, an in vivo assay was conducted using Gala apples with a diameter of 65–70 mm, produced in Hungary and obtained from a local supermarket. Each treatment was performed in triplicate (three apples per treatment). The apples were initially sterilized by immersion in a 2.5% sodium hypochlorite solution followed by 70% ethanol. The fungal suspension contained *A. clavatus* B9/6 at a concentration of 10^7^ conidia/mL, while the yeast suspension contained *W. anomalus* at 10^8^ CFU/mL. After the complete drying of the apple surface, 0.3 mL of inoculum was injected into each sample using a 0.7 × 30 mm sterile syringe. A negative control was included, in which only 0.9% saline solution was applied, along with a positive control, where only *A. clavatus* B9/6 was inoculated. Additionally, three experimental conditions were tested: (1) where the mold was inoculated 24 h before the yeast, (2) where the yeast was inoculated 24 h before *A. clavatus*, and (3) in which both microorganisms were inoculated simultaneously. The apples were then stored under ambient conditions, average temperature 24 °C; relative humidity not controlled, for 20 days. After this period, they were cut in half and disease severity was quantified from photographic images using ImageJ by calculating the proportion of lesion area relative to the total apple surface.

### Assessment of patulin removal by intracellular/extracellular enzymes and patulin binding of *W. anomalus*

*W. anomalus* 01499 was cultured in apple juice at 25 °C with constant agitation at 150 rpm until reaching 1 × 10⁸ CFU/mL, then centrifuged at 4,000 rpm for 40 min to separate the cells from the supernatant. The supernatant was filtered through a 0.22 μm membrane and stored at 4 °C for extracellular enzyme assays. The pellet was washed twice with 0.9% saline and divided for intracellular enzyme and cell wall adsorption analyses.

For extracellular enzyme evaluation, 10 mL of supernatant was incubated with patulin at 10 ppm at 25 °C for 24 h, followed by extraction and HPLC quantification.

For evaluation of intracellular enzymes, a portion of the cell pellet (adjusted to 1 × 10⁸ CFU/mL) was disrupted with a FRENCH^®^ Press at 850 psi, centrifuged at 4,000 rpm for 5 min to remove cell debris, and the resulting cell-free supernatant (5 mL) was incubated with patulin (10 ppm) at 25 °C for 24 h prior to HPLC analysis.

For evaluation of patulin adsorption by yeast cell walls, the method of Bata-Vidács et al. ^19^ was adapted for yeasts. Cell suspensions (1 × 10⁸ CFU/mL, 10 mL) were mixed with patulin (10 ppm) and incubated for 10 min at room temperature to allow toxin binding. Samples were then centrifuged at 4,000 rpm for 40 min to pellet the yeast cells together with any wall-bound patulin, and the supernatant containing unbound toxin was discarded. To quantify the adsorbed patulin, the pellet was extracted with 2 mL of dichloromethane by vortexing for 20 min in the dark, followed by centrifugation at 4,000 rpm for 10 min. The organic phase was collected, evaporated, and the residue resuspended in 1 mL of 10% acetonitrile prior to HPLC analysis.

### Patulin extraction

After centrifuging the apple juice contaminated with patulin, with or without yeast or molds at 4,000 rpm for 10 min, 4 mL of supernatant was transferred into a 15 mL Falcon tube, 2 mL of dichloromethane was added, and the tube was shaken vigorously and then left to stand in the dark for 20 min. One milliliter of the lower phase was pipetted into an Eppendorf tube and centrifuged at 4,000 rpm for 1 min, then 0.5 mL of the supernatant was pipetted into a clean Eppendorf tube. The dichloromethane was evaporated at 40 °C in a thermoshaker (BIOSAN TS-100) under a fume hood. The dried material was dissolved in 1 mL of 10% acetonitrile ^20^.

### High-performance liquid chromatography (HPLC) method

Patulin was quantified by HPLC under isothermal, isocratic conditions using a Kinetex 2.6 μm XB-C18 100 Å column (150 × 4.6 mm). The mobile phase was acetonitrile–water (10:90, v/v) at 0.3 mL/min, with UV detection at 276 nm. The retention time of patulin under the described isocratic HPLC conditions was 4 min. The limit of detection (LOD) for the method was 3 µg/kg, determined based on a signal-to-noise ratio of 3 ^20^.

### Statistical evaluation

All experiments were conducted in triplicate. Statistical analyses were performed using STATISTICA 7.0 software, with significance set at *p* < 0.05. Differences between groups were assessed using ANOVA and Tukey’s test for multiple comparisons, where applicable. Data are presented as mean ± standard deviation (SD), and error bars in the figures represent SD.

## Results

### Screening of yeast strains for antifungal activity

Twenty different commercial yeast products were tested for their potential to inhibit *A. clavatus* B9/6 in a preliminary screening, but none of the tested yeasts exhibited antifungal activity. *A. clavatus* B9/6 continued to grow uninhibited, eventually spreading over the yeast colonies in the Petri dishes. Similar results were observed for the *Saccharomyces* species obtained from the NCAIM collection, where no inhibition of *A. clavatus* B9/6 was detected in a preliminary screening.

**Fig. 1:**
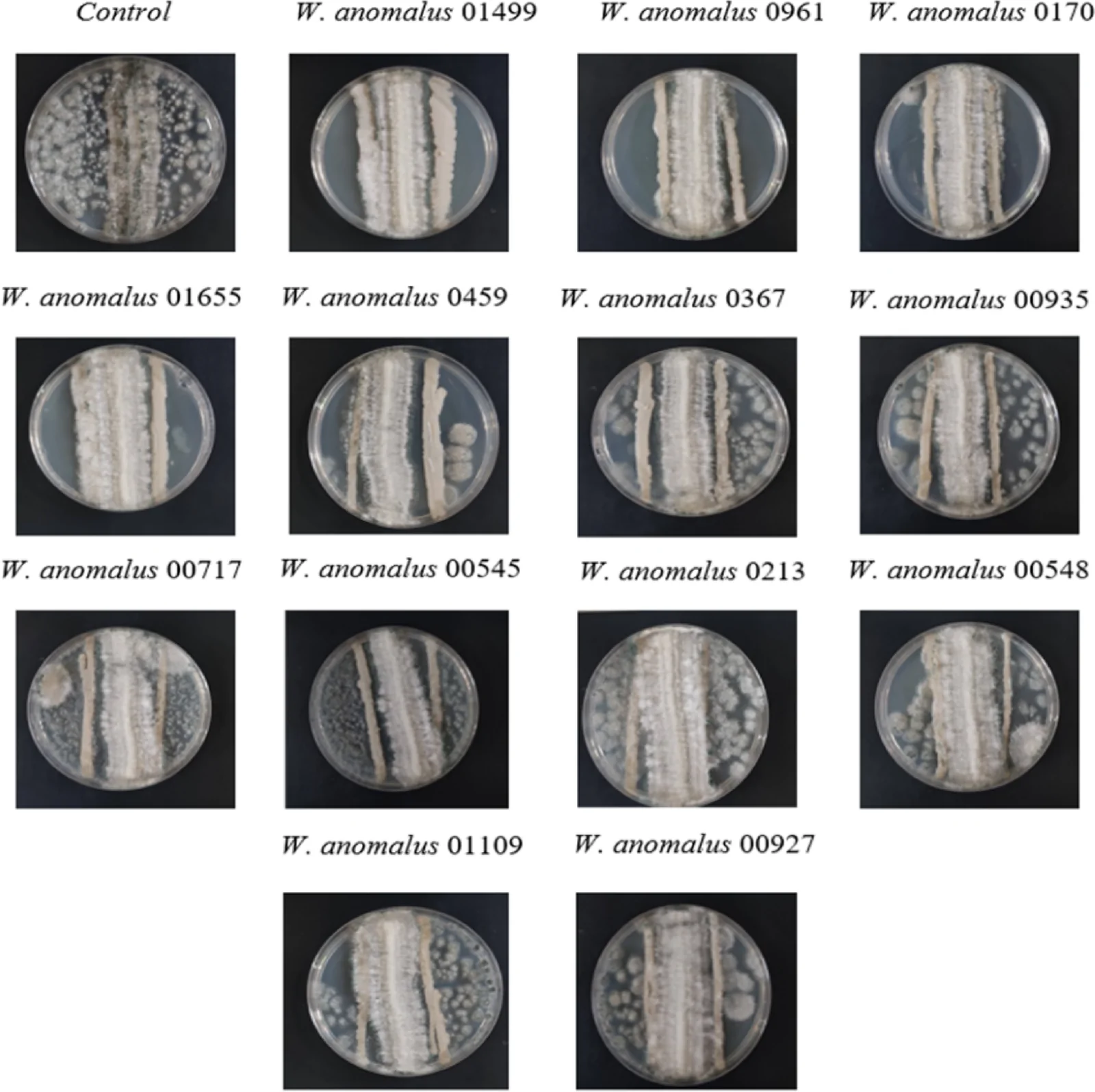
Semi-qualitative confrontation assay illustrating the ability of different *W. anomalus* strains to restrict the spatial spread of *A. clavatus* B9/6 on malt extract agar after 7 days at 25 °C.

On the other hand, all thirteen (13) strains of *W. anomalus* obtained from NCAIM showed visible inhibition zones around yeast colonies when tested on PDA medium containing *A. clavatus* B9/6 spores (Supplementary Fig. S1). Therefore, a second experiment was carried out with the *W. anomalus* yeasts, in order to evaluate the ability of the yeasts to restrict the spatial spread of *A. clavatus* from a defined inoculation line, as illustrated in Fig. 1.

To reduce subjectivity in strain selection, the visual observations obtained from the confrontation assay were classified using a semi-quantitative scoring system based on the number of fungal colonies detected outside the inoculation zone. The resulting classification is presented in Table 2.

**Table 2:** Semi-quantitative classification of the antifungal activity of *W. anomalus* strains against *A. clavatus* B9/6 based on colony development outside the inoculation zone after 7 days of incubation.

Score	Criteria	Strains
3	No colonies detected beyond the inoculation zone	*W. anomalus* 01499, 0961, 0170, 01655
2	1–2 colonies detected beyond the inoculation zone	
1	3–5 colonies detected beyond the inoculation zone	*W. anomalus* 0459
0	More than 5 colonies detected beyond the inoculation zone	*W. anomalus* 0367, 00935, 00717, 00545, 0213, 00548, 01109, 00927

Four strains, *W. anomalus* 01499, 0961, 0170, and 01655, completely prevented the appearance of colonies outside the defined growth area under the experimental conditions and therefore received the maximum score (3). *W. anomalus* 0459 showed partial restriction of fungal spread and received a score of 1. The remaining strains exhibited more than five colonies outside the inoculation zone and were classified with a score of 0. Based on this classification, strains 01499, 0961, 0170, and 01655 were selected for subsequent patulin removal assays.

### Patulin removal by yeasts

**Fig. 2:**
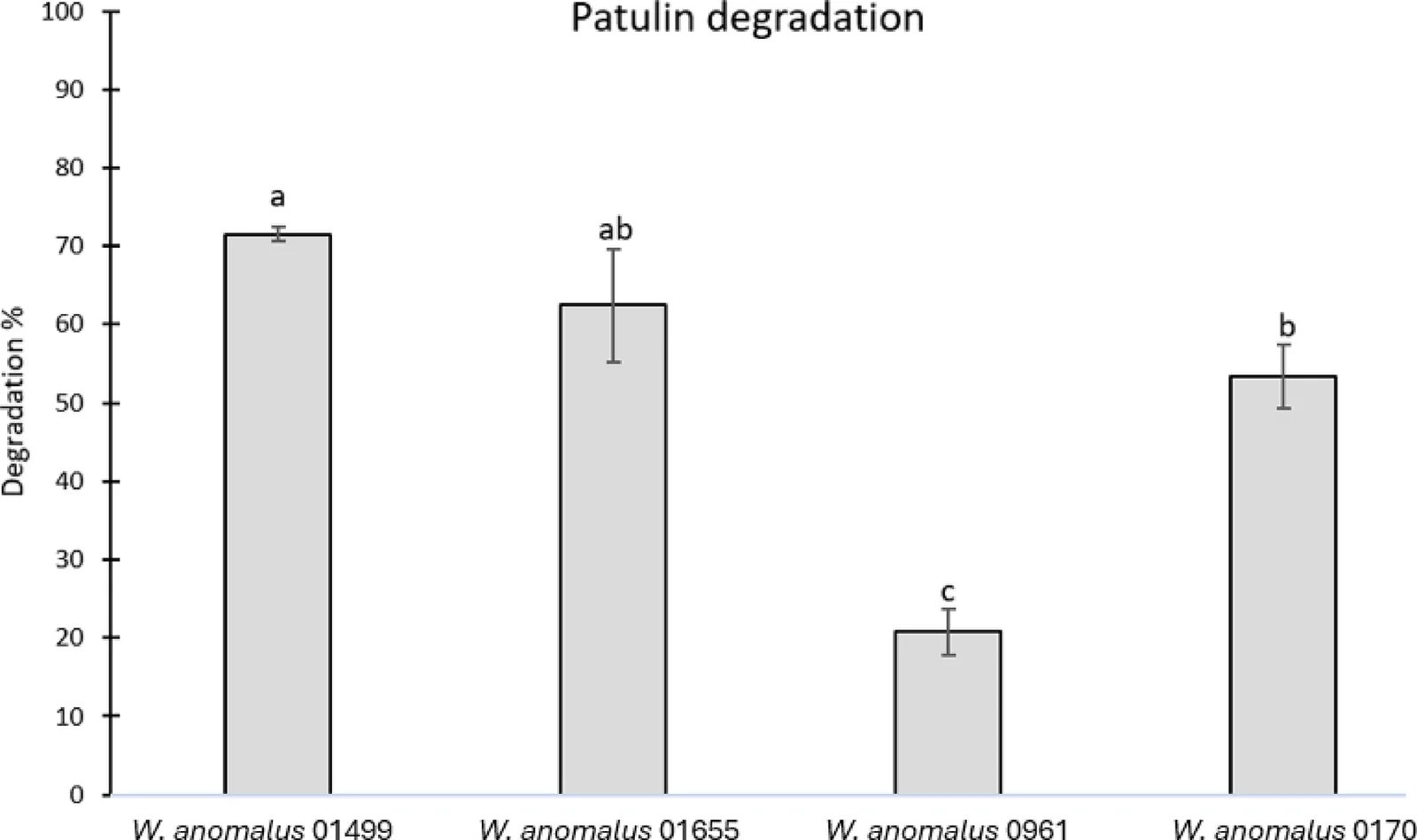
Patulin degradation by different strains of *W. anomalus* in apple juice at 25 °C. Bars represent mean ± SD (*n* = 3). Different letters indicate significant differences according to Tukey’s test (*p* < 0.05).

To evaluate the removal of patulin (PAT), an assay was conducted using *W. anomalus* 01499, 01655, 0961, and 0170 in apple juice. Figure 2 shows the percentage of PAT removal for each of the four strains studied after 24 h of incubation at 25 °C with an initial patulin concentration of 10 ppm.

Among them, *W. anomalus* 01499 showed the highest PAT removal at 71% after 24 h, followed by *W. anomalus* 01655 (62%) and *W. anomalus* 0170 (53%). The lowest PAT removal was observed for *W. anomalus* 0961 at 20%. No significant decrease in PAT levels was observed in the control after the 24 h incubation. A one-way ANOVA revealed a significant effect of yeast strain on patulin removal, F(3, 8) = 50.37, *p* = 0.00124.

### Kinetics of patulin removal

**Fig. 3:**
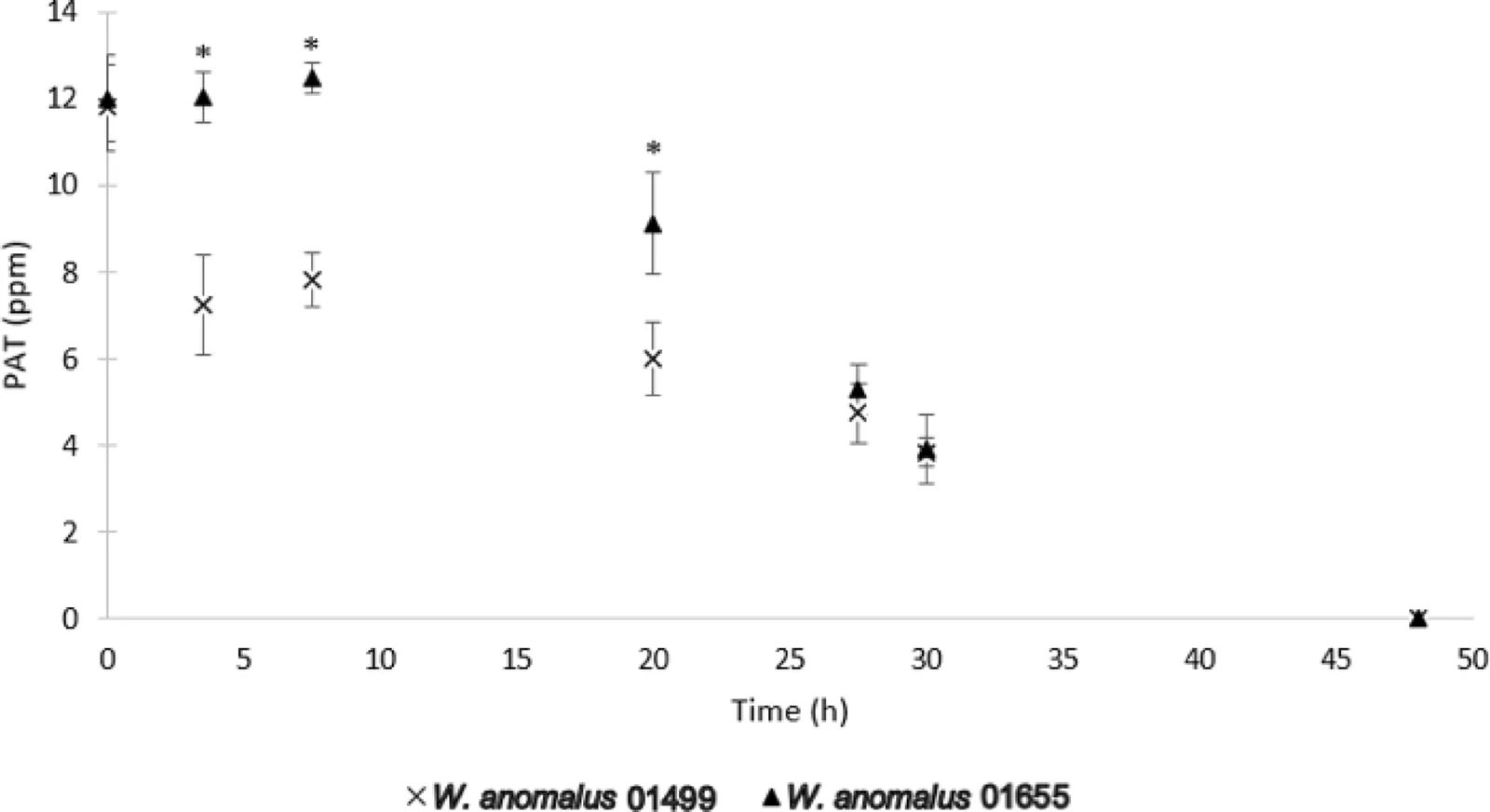
Patulin removal kinetics by *W. anomalus* 01499 and 01655 in apple juice at 25 °C. Symbols represent mean ± SD (*n* = 3), *indicates *p* < 0.05 between strains at the same time point (Tukey’s test).

The kinetics of PAT removal were studied for *W. anomalus* strains 01499 and 01655. Since complete PAT removal was not achieved within 24 h, the incubation period was extended to 48 h. Figure 3 illustrates the changes in toxin concentration over time when incubated with viable yeast cells in apple juice at 25 °C.

Both strains evaluated were able to remove the total amount of PAT during 48 h under the studied conditions; however, the profiles of PAT removal were different for the two strains. Although the nominal initial concentration was 10 ppm, the actual patulin levels were determined analytically by HPLC, and slight variations reflect experimental and analytical variability; all calculations were based on the measured control values. In the first 10 h of the experiment, no significant change in the patulin content was observed for *W. anomalus* 01655, the concentration started to fall from the 20th hour and presented a declining linear profile until reaching its lowest value at 48 h. For the strain *W. anomalus* 01499, in the first 4 h of the experiment, a reduction in the concentration of patulin of approximately 35% was already observed, but then, until 10 h, no significant change was noted. Between hours 10 and 20, the concentration started to decrease again, but in a less abrupt manner than for 01655. From the 20th hour, a slow decrease was observed until total removal of the mycotoxin by the end of the experiment. No significant decrease in PAT level was observed in the control. A two-way ANOVA showed significant effects of time (*F*(6, 21) = 78.14, *p* < 0.0001), group (*F*(1, 21) = 38.30, *p* < 0.0001), and time × group interaction (*F*(6, 21) = 6.42, *p* = 0.00059), indicating that the two curves had different removal patterns over time.

### In vivo evaluation of *A. clavatus* B9/6 inhibition using *W. anomalus*

**Fig. 4:**
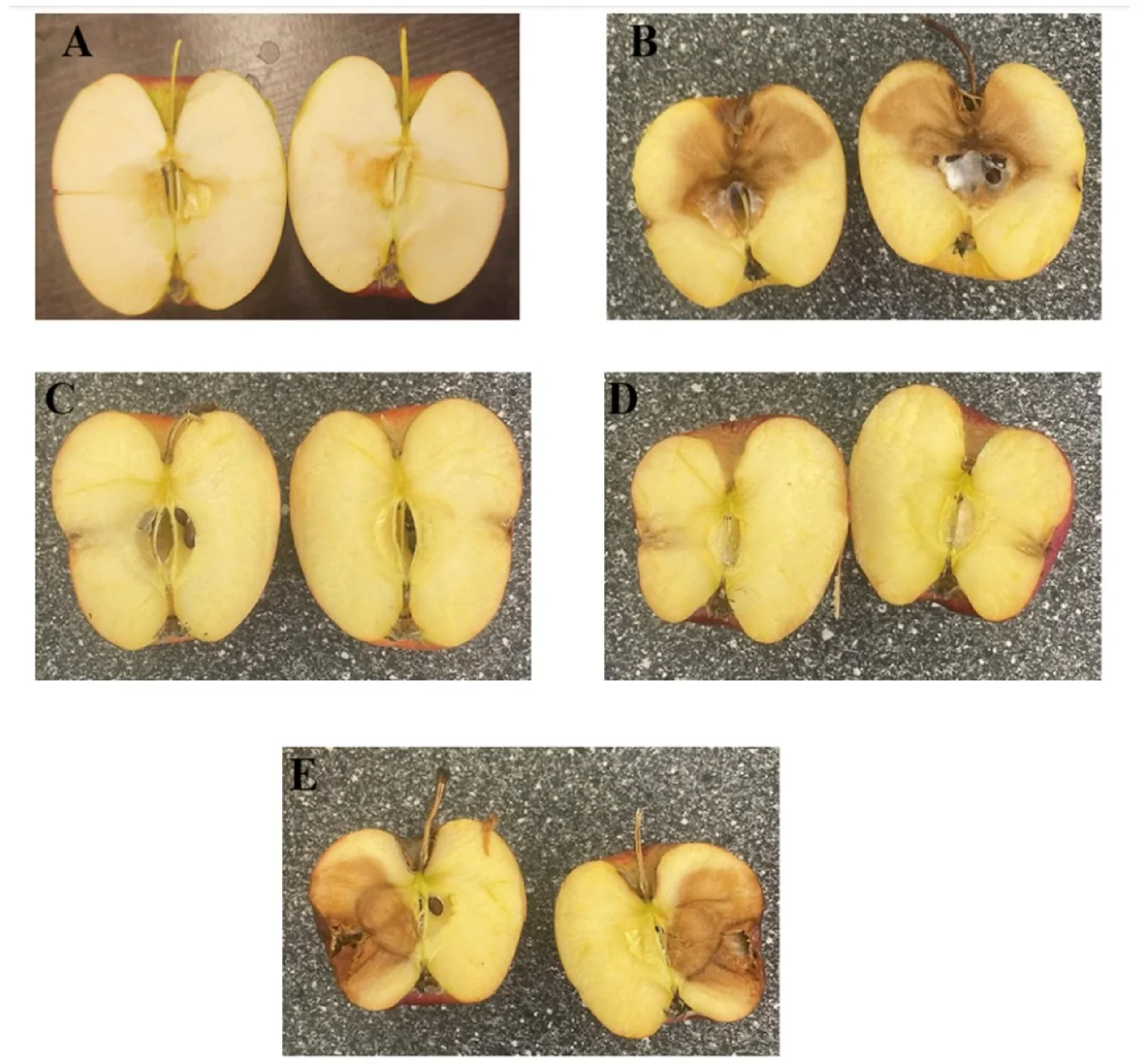
In vivo assay of inhibition of *A. clavatus* B9/6 using *W. anomalus* 01499. (**A**) Negative control, (**B**) Positive control, (**C**) *W. anomalus* 01499 injected 24 h before *A. clavatus* B9/6, (**D**) *W. anomalus* 01499 injected at the same day as *A. clavatus* B9/6, (**E**) *A. clavatus* B9/6 injected 24 h before *W. anomalus* 01499.

To evaluate the potential of *W. anomalus* 01499 to inhibit *A. clavatus* B9/6 in actual fruits, tests were performed by inoculating both microorganisms at different times. The results are presented in Fig. 4.

Image-based quantification of infected tissue showed that the positive control exhibited 44.2% lesion coverage, whereas apples treated with *W. anomalus* 24 h prior to fungal inoculation showed only 0.8% affected tissue. Simultaneous inoculation resulted in 2.5% lesion coverage, while inoculation of the yeast after the mold resulted in 36.6% lesion coverage.

### Patulin removal by extracellular metabolites, intracellular extracts, and cell wall adsorption in *W. anomalus* 01499

To investigate the mechanisms involved in patulin (PAT) removal by *W. anomalus* 01499, the contributions of extracellular metabolites, intracellular extracts, and cell-wall-associated adsorption were evaluated separately. The results are summarized in Table 3.

**Table 3:** Patulin removal by extracellular metabolites, intracellular extracts, and cell wall adsorption in *W. anomalus*.

Fraction tested	Residual PAT (ppm)	Cell associated PAT recovered (ppm)	Removal (%)
Control (PAT only)	10.73 ± 0.40^a^	–	–
Extracellular supernatant	11.26 ± 0.86^a^	–	0
Intact cells (adsorption assay)	–	1.18 ± 0.18*	11.0
Intracellular extract	7.23 ± 0.12^b^	–	32.6

Removal (%) for intracellular and extracellular fractions was calculated from the decrease in soluble PAT concentration relative to the control. *For yeast cells, the reported value represents the proportion of the initial PAT recovered from the cell-associated fraction after solvent extraction, indicating adsorption of the toxin onto the cell surface.

Values represent mean ± SD (*n* = 3).

Different letters (a, b) indicate significant differences according to Tukey’s test (*p* < 0.05).

For intracellular and extracellular fractions, PAT reduction was calculated from the decrease in soluble toxin concentration relative to the control. For the cell wall fraction, PAT removal reflects adsorption of the toxin onto wall components, quantified by solvent extraction of the bound fraction.

No decrease in PAT concentration was observed in the extracellular supernatant, indicating that soluble metabolites released into the medium did not contribute to toxin removal under the tested conditions. Slight variations above the nominal initial concentration (10 ppm) are attributed to analytical variability and recovery efficiency of the HPLC method.

In contrast, yeast cells demonstrated the ability to bind PAT, as confirmed by solvent extraction and quantification of the toxin associated with the cell pellet. Approximately 11% of the initial PAT content was recovered from the cell-associated fraction following extraction, indicating adsorption of the toxin onto cell surface components.

The intracellular extract produced the greatest decrease in PAT concentration, resulting in approximately a 33% reduction relative to the control. Since this fraction may contain a mixture of intracellular enzymes, proteins, and other cellular constituents, the observed effect is attributed to intracellular components collectively rather than to specific enzymatic activity alone.

## Discussion

Yeast-based biocontrol solutions have been extensively studied across various fields. The genus *Saccharomyces* plays a crucial role in the food industry, particularly for bakery products and fermented beverages, and is classified as generally recognized as safe (GRAS). Previous research has explored the potential of *Saccharomyces* species in patulin (PAT) detoxification, yielding promising results. For instance, Li et al. ^21^ demonstrated that *S. cerevisiae* CITCC 93,161 could degrade 100% of PAT at a concentration of 100 mg/L. Additionally, the YKL069W enzyme of *S. cerevisiae* CICC 31,084 was identified as capable of binding and degrading PAT into E-ascladiol within 48 h at an initial concentration of 10 µg/mL ^22^.

Twenty commercial yeast products, primarily *S. cerevisiae* and related species widely used in beer, wine, and cider production, were evaluated for their ability to inhibit *A. clavatus* B9/6. These strains were selected for their broad industrial use, which would facilitate large-scale application if antifungal activity were detected. However, none inhibited *A. clavatus* B9/6, which grew uninhibited and eventually overgrew the yeast colonies, indicating the absence of antifungal properties under the tested conditions.

Given these results, further experiments were conducted with *W. anomalus*, a GRAS-classified species with potential applications in the food industry. Previous studies have demonstrated the biocontrol potential of *W. anomalus* against fungal contaminants such as *P. roqueforti*, *A. candidus*, *P. italicum*, and *A. flavus *^23–26^. However, its effectiveness against *A. clavatus* has not been previously explored. The antifungal activity of *W. anomalus* is primarily attributed to its production of volatile organic compounds (VOCs) and, in some cases, killer toxins ^27,28^.

All 13 strains tested in this study showed some level of inhibition against *A. clavatus* B9/6; therefore, *W. anomalus* strains 01499, 01655, 0961, and 0170, which showed the best inhibition results, were selected for further experiments to evaluate their ability to not only inhibit the mold but also remove PAT in apple juice. This dual approach is critical, as inhibition alone may not mitigate existing toxin levels, whereas degradation/removal directly reduces patulin concentration, thereby improving the safety and quality of the juice. The use of a real food matrix was based on Ma et al. ^29^, who reported that PAT degradation varies between culture media and fruit juices due to nutrient composition, with degradation in pear juice taking 12 h longer than in NYBD medium.

Various microorganisms have demonstrated the ability to degrade PAT in different fruit matrices. For instance, *Hannaella sinensis* achieved approximately 70% PAT removal in pear juice after 24 h ^29^, while *Lactobacillus pentosus* (DSM 20314) reached 53% in apple juice, alongside other lactic acid bacterium species with varying efficiencies ^30^. Similarly, *Rhodosporidium paludigenum* reduced PAT levels by 70% using viable cells and 50% with inactivated cells ^31^.

In comparison, *W. anomalus* strains tested in this study showed notable PAT removal capacities, with strain 01499 outperforming previously studied microorganisms by reducing PAT by 71% after 24 h. Strains 01655 and 0170 followed with 62% and 53% PAT removal, while 0961, though less effective, achieved 20%. These results highlight *W. anomalus* as a promising candidate for PAT detoxification, with 01499 being particularly efficient. The initial patulin concentration used in this study (10 ppm) was substantially higher than the maximum regulatory limit established for apple juice. Nevertheless, concentrations in the mg/L range have frequently been used in microbial patulin degradation studies to enable reliable analytical quantification and kinetic evaluation ^12,21^. As the effect of initial toxin concentration on removal efficiency was not investigated, further studies should assess the performance of *W. anomalus* under contamination levels more representative of commercial products.

Microbial degradation of PAT generally detoxifies the toxin by forming intermediates such as (E)- and (Z)-ascladiol or hydroascladiol, which are far less toxic. Zheng ^32^ showed that *Pichia caribbica* degradation produced ascladiol and other products with minimal toxicity to bacteria, plants, and human epithelial cells. Similarly, *Lactobacillus casei* YZU01 degradation products caused no liver or intestinal damage in mice and did not alter gut microbiota ^13^, supporting the conclusion that PAT biodegradation yields products with negligible toxicological impact.

In in vivo tests, *W. anomalus* 01499 effectively controlled *A. clavatus* growth, likely due to its rapid colonization and competition for nutrients. This supports previous findings suggesting that yeast dominance restricts fungal development and toxin production ^25,33^. In contrast, the positive control, where only *A. clavatus* was introduced, exhibited severe fruit decay, characterized by dark lesions and extensive mycelial growth. When *Aspergillus* was inoculated 24 h before the yeast, disease symptoms were significantly more severe, likely because the fungus had already established infection and colonized the fruit tissue. This early colonization weakens physical barriers and natural defenses, creating favorable conditions for virulence expression and potentially enhancing patulin production. Similar observations have been reported by Zhu et al. ^31^ for *Rhodosporidium paludigenum*, where enhanced fungal virulence correlated with elevated patulin production. Therefore, the increased virulence observed in this temporal inoculation sequence likely results from the early fungal colonization, leading to more pronounced disease symptoms.

The mechanisms of PAT removal by *W. anomalus* were examined by assessing the contributions of extracellular enzymes, intracellular enzymes released upon cell disruption, and PAT adsorption to the yeast cell wall. Supernatant assays showed no effect, suggesting the absence of secreted enzymes under the tested conditions, consistent with *H. sinensis* where no PAT degradation occurred after 30 h ^29^. Cell wall assays showed an 11% reduction, indicating partial adsorption, unlike *H. sinensis*, but aligning with *S. cerevisiae*, where heat-treated cells increased mitigation ^34^. Intracellular components (including enzymes) played the main role, reducing PAT by 33%, and higher rates may be possible under optimized conditions. Future research should focus on optimizing conditions such as pH, temperature, and nutrient availability to enhance enzymatic activity and PAT removal. Additionally, adaptive pre-exposure to PAT may stimulate higher enzymatic activity, as demonstrated in *H. sinensis *^29^. Strategies such as metabolic engineering and extended fermentation could enhance the detoxification efficiency of *W. anomalus*, reinforcing its potential as a biocontrol agent against mycotoxin contamination.

While previous studies have demonstrated patulin removal by different microorganisms, the present results extend these observations to *W. anomalus* strains exhibiting both antifungal activity against *A. clavatus* and patulin removal capacity. In addition, the mechanistic assays suggest that intracellular components contribute more substantially to patulin removal than extracellular factors or cell wall adsorption, providing a basis for further investigation of the detoxification process.

In this study, *W. anomalus* strains showed a strong ability to control *A. clavatus* and reduce patulin levels in apples. Their combined inhibition of fungal growth and toxin removal, mainly *via* intracellular enzymes and cell wall binding, underscores their value as dual-purpose agents for post-harvest protection and food safety. Identification of patulin degradation products (e.g., ascladiol) was beyond the scope of the present study and should be addressed in future work to better characterize the detoxification pathway.

Since antifungal efficacy may vary among fungal species and strains, future studies should evaluate the performance of *W. anomalus* against other *A. clavatus* strains and additional patulin-producing fungi, particularly *P. expansum*, which is the major cause of patulin contamination in apples.

## Supplementary Information

Below is the link to the electronic supplementary material.

Supplementary Material 1

## Data Availability

All data generated or analyzed during this study are included in this published article.
