# Supplementary material for: Wickerhamomyces anomalus as a biocontrol agent against Aspergillus clavatus and patulin in apples
Source: Sci Rep. 2026 Jun 19;16:28193. doi: 10.1038/s41598-026-57297-5 (PMC13554278; doi:10.1038/s41598-026-57297-5)
Supplement: Supplementary file 1 [file 41598_2026_57297_MOESM1_ESM.docx]

**Supplementary Figure S1. Representative inhibition assay of *A. clavatus* B9/6 on PDA medium.** (A) Control plate inoculated only with *A. clavatus* B9/6. (B) Inhibition zone produced by *W. anomalus* 01499. (C) Inhibition zone produced by *W. anomalus* 01109. (D) Reverse side of the plate inoculated with *S. willianus*. (E) Front view of the plate inoculated with *S. willianus*, showing absence of inhibition.
